# Dissecting the regulation of EBV’s BART miRNAs in carcinomas

**DOI:** 10.1016/j.virol.2017.02.013

**Published:** 2017-03-01

**Authors:** Ya-Chun Yang, Amy Liem, Paul F. Lambert, Bill Sugden

**Affiliations:** McArdle Laboratory for Cancer Research, University of Wisconsin-Madison, 1111 Highland Avenue, Madison, WI 53705-2275, United States

**Keywords:** Epstein-Barr virus, Micro RNAs, BamHI A rightward transcripts

## Abstract

Epstein-Barr virus (EBV) encodes multiple miRNAs known to contribute to its pathogenicity. Previous studies have found that the levels of some EBV miRNAs are 10–100 fold higher in biopsies and in tumor xenografts than in cells grown in culture. We have asked if these increased levels reflect transcriptional enhancement resulting from the tumor microenvironment, selection for increased levels of the EBV genome, or both. We measured the levels of BART miRNAs and their DNA templates in tumor xenografts induced from EBV-positive gastric carcinoma cells and EBV-negative gastric carcinoma cells expressing plasmid replicons encoding these miRNAs. We focused on BART miRNAs which are expressed in all tumors and found that they provide tumors selective growth advantages as xenografts. Stem-loop PCR and real-time PCR revealed that the xenografts expressed both higher levels of some miRNAs and viral DNA templates than did the corresponding cells in culture.

## 1. Introduction

Epstein-Barr virus (EBV) is a human herpesvirus that successfully infects more than 90% of the human population (Williams and Crawford, 2006). Infection with EBV is associated with several human cancers, including Burkitt’s lymphoma (BL), Hodgkin’s disease, post-transplant lymphoproliferative disease, and both gastric and nasopharyngeal carcinoma (NPC) (Brady et al., 2007; Hammerschmidt and Sugden, 2004; Raab-Traub, 2002, 2015; Sugden, 2014; Tse and Kwong, 2015). This tumor virus infects quiescent B cells, induces their proliferation, and is maintained in them in a latent state where its genome exists as a circular plasmid. Defects in EBV’s DNA synthesis lead to its being lost from a population of proliferating cells unless it provides the cells that retain it sufficient selective advantages to outgrow those that lose it (Kennedy et al., 2003; Nanbo et al., 2007; Vereide and Sugden, 2009). Multiple studies have shown that EBV’s miRNAs provide selective advantages early in infection of B-cells and to B-cell tumors (Albanese et al., 2016; Bernhardt et al., 2016; Feederle et al., 2011; Tagawa et al., 2016; Vereide et al., 2014).

EBV encodes 25 pre-miRNAs of which 3 are in the BHRF locus and 22 in the BART locus. Their processed, mature miRNAs have been shown to regulate both cellular and viral functions (Barth et al., 2011; Cai et al., 2006; Kuzembayeva et al., 2014). The BART cluster is expressed in all EBV-positive proliferating cells examined (Pfeffer et al., 2004; Pratt et al., 2009). The BART miRNAs foster EBV’s life-cycle by inhibiting apoptosis and immune responses to infected B-cells (Albanese et al., 2016; Lin et al., 2015; Tagawa et al., 2016; Vereide et al., 2014). The BART miRNAs also likely promote epithelial cell survival by targeting multiple pro-apoptotic cellular genes that could contribute to the EBV-mediated epithelial carcinogenesis (Cai et al., 2015; Kanda et al., 2015; Kang et al., 2015; Marquitz et al., 2014, 2012; Shinozaki-Ushiku et al., 2015). Most of EBV’s miRNAs are processed from two adjacent regions separated by one copy of oriLyt in the BART locus. They are located in the first four introns of its long primary transcript and regulated by two alternative promoters, P1 and P2 (Chen et al., 2005). Although BART miRNAs are transcribed from a single primary transcript, the expression patterns of BART miRNAs vary dramatically among different EBV-positive cell lines (Hooykaas et al., 2016; Pratt et al., 2009; Yang et al., 2013).

The expression of these miRNAs has been examined both in biopsies of EBV-positive tumors and in cells derived from them in cell culture. One surprising observation from these studies is that EBV’s miRNAs are expressed at higher levels in carcinoma biopsies than in cells grown in culture derived from them (Chen et al., 2010; Qiu et al., 2015). These studies have been replicated in experiments in which carcinoma cells have been grown as tumor xenografts in immunocompromised mice. The levels of the BART miRNAs were found to be 10–100 fold higher in the xenografts than in the cells grown in parallel in cell culture (Qiu et al., 2015). These surprising findings are particularly intriguing because higher levels of expression of miRNAs would increase the range of mRNAs they target and thus their impact on the infected tumor cells. There are at least two mechanistic explanations for increased expression of the BART miRNAs, which we have dissected here. In one, the tumor microenvironment could elicit enhanced transcription and/or processing of these miRNAs. In the second, selection for miRNA function in the tumors could lead to an increase in copy number of the viral plasmids that are their transcriptional templates to yield the higher levels of detected BART miRNAs. We have used multiple xenografts to determine whether one, the other, or both of these mechanisms underlie the observed differential expression of the viral miRNAs.

We used two cell lines to examine the expression of EBV miRNAs in cell culture and in tumors that form from them as xenografts in Hsd:Athymic Nude-Foxn1^nu^ mice. SNU-719 cells are EBV-positive and derived from a gastric carcinoma (Oh et al., 2004). AGS cells are derived from an EBV-negative gastric carcinoma (Barranco et al., 1983). Plasmid vectors derived from EBV that express either all of EBV’s BART miRNAs, a subset of them, or no miRNAs were introduced into the AGS cells in order to determine their expression without the influence of other EBV genes. The levels of representative viral miRNAs were measured in these cells propagated in culture and in the tumors they formed when grown as xenografts in immunoincompetent mice. Different numbers of cells were used as inocula so that the times for tumor development ranged from one to three and one-half months and thus allowed different times for expression of the selective advantages the miRNAs might afford the tumors. Overall we analyzed 76 tumors derived from these two cell lines for their expression of EBV miRNAs and compared those levels to that in the parental cell lines grown in culture.

## 2. Materials and methods

### 2.1. Cells

EBV-positive SNU-719 cells (Oh et al., 2004) and EBV negative AGS cells (Barranco et al., 1983) derived from gastric carcinomas, were cultured in RPMI 1640 (Invitrogen) supplemented with L-glutamine, 10% fetal bovine serum (FBS), and antibiotics (200 U/ml penicillin and 200 μg/ml streptomycin).

AGS cells were transfected with different plasmid DNAs using Lipofectamine 2000 (Invitrogen) as described by the manufacturer. The cells were selected with hygromycin, clones were isolated, and characterized using real time PCR. AGS-3829, AGS-4045 and AGS-4046 are stably transfected with plasmids p3829, p4045 and p4046, respectively. AGS-NC are AGS cells that stably express either p3828, or p3828 containing luciferase.

### 2.2. Plasmids

Plasmids were derived from OriP vectors and contained either no insert (p3828) or the intact promoter for the BART miRNAs, the BART-miRNA coding region, and an SV40 polyA addition sequence (p3829). They were constructed by David Vereide (Vereide, University of Wisconsin, Madison, 2009). Plasmids p3828 and p3829 were used previously in studies of miRNA expression in xenografts (Qiu et al., 2015). Two derivatives of p3829 were made that delete the DNA in the BART locus encoding either miRNAs BART 15–17 and 5–6 (p4045) or miRNAs BART 7–14 and 18–22 (p4046).

### 2.3. Mice

Female Hsd:Athymic Nude-Foxn1^nu^ mice (ENVIGO) ages 6–8 weeks were housed and maintained under sterile conditions with food and water as prescribed by AAALAC. All mouse procedures were performed according to a protocol approved by the Research Unit for Laboratory Animal Care Committee. Mice were injected subcutaneously at 4 sites with either 1×10^5^ cells or 5×10^6^ cells resuspended in 100 μl phosphate-buffered saline (PBS) and mixed with an equal volume of Matrigel (Topley et al., 1993). The mice were followed for 1–3.5 months, and palpable tumors less than 1 cm in diameter collected for analysis.

### 2.4. Total RNA and genomic DNA isolation

50–100 mg of tumor tissue was homogenized using a Dounce homogenizer with 1 ml TRIzol (Ambion); after phase separation by extraction with 0.2 ml chloroform, the aqueous phase was recovered for RNA extraction and the interphase and phenol-chloroform phase were used for isolation of genomic DNA. Total RNA and genomic DNA were extracted according to the manufacturer’s instruction. Briefly, RNA was precipitated by adding an equal volume of 100% Isopropanol with 5 μg/ml linear acrylamide to the aqueous phase of TRIzol mixture. The RNA pellet was then washed with 75% ethanol and treated with DNAase (QIAGEN) to remove contaminating DNA. Genomic DNA was precipitated by adding 0.3 ml 100% ethanol to phenol-chloroform phase of TRIzol mixture. Then the DNA pellet was washed by citrate/ethanol solution (0.1 M sodium citrate in 10% ethanol, pH8.5). After washing, the DNA pellet was dissolved in 8 mM NaOH and then adjusted to pH7-8 with HEPES.

### 2.5. Stem-loop real-time PCR

For each miRNA assayed, 200 ng of total RNA was reversed transcribed using TaqMan MicroRNA Reverse Transcription Kit (Applied Biosystems) as described by the manufacturer. Each 20 μl real-time PCR reaction contained 1.5 μM forward primer, 0.7 μM reverse primer, 0.2 μM probe, and 1X TaqMan Universal Master Mix, no UNG AmpErase (Applied Biosystems) was used as described previously (Pratt et al., 2009).

## 3. Results

### 3.1. Expression of BART miRNAs in SNU-719-induced tumor xenografts and in cultured SNU-719 cells

**Fig. 1:**
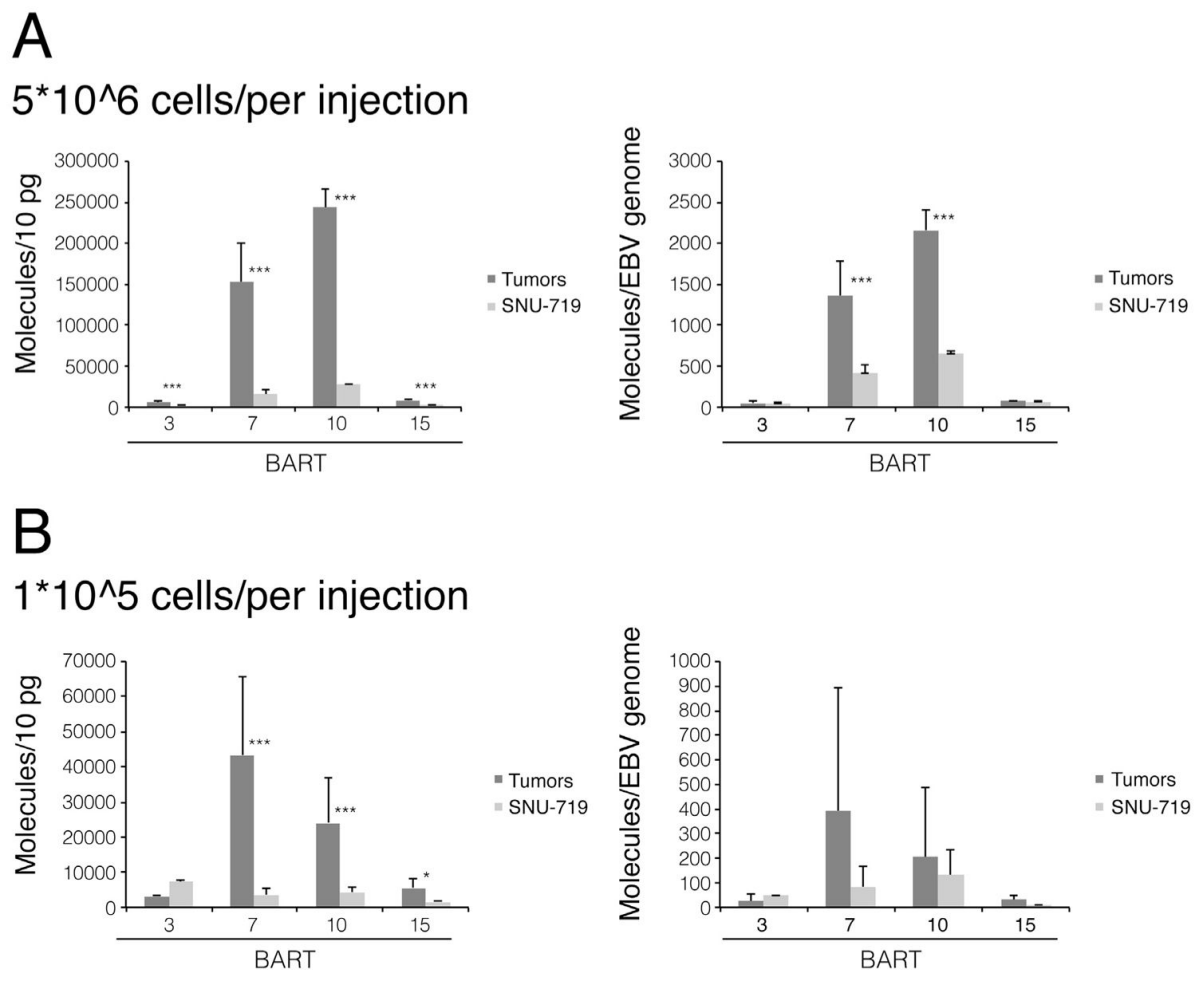
Some BART miRNAs expressed in SNU-719-induced tumor xenografts and in cultured SNU-719 cells are expressed differently independent of the tumor inocula. 5×10^6^ (A) or 1×10^5^ (B) SNU-719 cells were injected into mice to induce tumors. The tumors were collected and total RNA and DNA were isolated. Four BART miRNAs were measured by stem loop real-time PCR. The level of each miRNA was calculated by generating standard curves with known quantities of synthetic miRNA identical to the mature miRNA sequence. In addition, each BART miRNA expression level was normalized to the number of EBV genomes measured by real-time PCR. All miRNAs were measured in more than three independent experiments. Statistical analysis with Student’s *t*-test was performed to compare the miRNA expression levels in tumor xenografts and in cultured cells. Error Bars represent the standard deviation. *, p < 0.05; ***, p < 0.0005.

SNU-719 cells (5×10^6^ cells) were injected subcutaneously at four sites each into two nude mice. Palpable tumors arose at all eight tested sites in one month (Table 1) and ranged from 0.5 to almost 1 cm in diameter. The tumors were harvested and both DNA and RNA isolated from them. In general, 7.5×10^4^–1.2×10^5^ ng of RNA was recovered from 1×10^7^ tumor cells or their parental cells grown in culture indicating that we recovered between 7.5 and 12 pg of RNA per cell. Stem-loop real-time PCR was used to measure the levels of four miRNAs in these cells, BART3, BART7, BART10, and BART15. BART3 and 15 are encoded in the first portion of the BART transcript upstream of oriLyt while BART 7 and 10 are encoded in the second portion of miRNAs downstream of oriLyt. Standard curves with known quantities of synthetic miRNAs identical to the mature miRNAs were used for quantifying miRNA expression levels and are given per 10 pg of total cellular RNA, the average amount of total RNA recovered per cell. In general the levels of EBV miRNAs were higher in the tumor cells than in their parental cells grown in culture with the increases ranging from 3 to 9-fold (Fig. 1A). However, measurements of viral DNA per cell with real time PCR demonstrated a 2.9-fold increase of EBV DNA copy number in the SNU-719 tumor cells relative to those cells grown in culture (Table 2). We normalized the measured levels of each of the miRNAs to that of the viral plasmid templates per cell and found that the expression of BART7 and BART10 were 3-fold higher in tumor xenografts than in cultured cells, while that of BART3 and BART15 were the same (Fig. 1A).

**Fig. 2:**
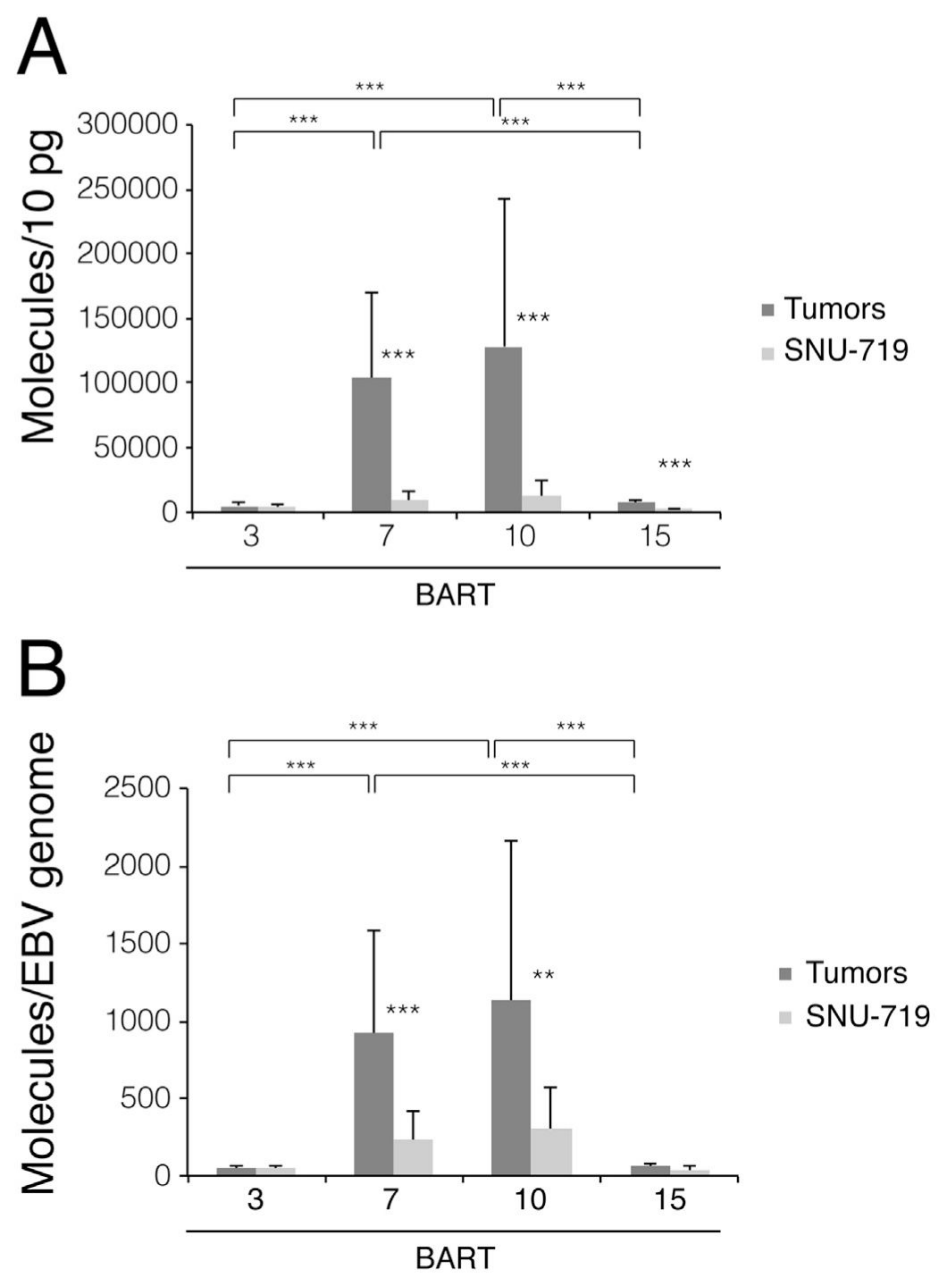
BART7 and BART10 accumulate to higher levels in SNU-719-induced tumor xenografts than in their parental cells grown in culture. (A) The measurements for BART miRNAs in Fig. 1A and B were pooled and analyzed as were their normalized values (B). Statistical analysis with Student’s *t*-test was performed to compare the miRNA expression levels in tumor xenografts and in cultured cells, and expression levels between different BART miRNAs. Error Bars represent the standard deviation. *, p < 0.05; **, p < 0.005; ***, p < 0.0005.

We tested whether the small differences of miRNA expression between xenografts and cells in culture might reflect the short period of tumor growth being insufficient to allow any selective advantages of the miRNAs to be manifested. Therefore, the inoculum was reduced to 1×10^5^ SNU-719 cells which took 3.5 months to grow to palpable tumors. Again 8 injections yielded 8 tumors (Table 1). Measurements of the miRNAs indicated that the expression of BART7 increased 12-fold, that of BART10 and BART15 increased 5.5-fold and 3.5-fold respectively, while that of BART3 decreased slightly in the tumor cells relative to the cultured cells (Fig. 1B). Normalizing these measurements to the EBV plasmid copy number, lowered the increases of BART7, BART10, and BART15 miRNAs to 4.7-fold, 1.6-fold, and 3-fold per DNA template, respectively (Fig. 1B). These experiments show that extending the time to tumor development with EBV-positive cells does not select for increases in expression of EBV’s miRNAs per DNA template. When all of the measurements for the xenografts were considered and normalized per DNA template, the levels of BART7 and 10 were 4-fold higher in the tumors than in the cells in culture while those of BART3 and 15 were the same in both cell types (Fig. 2A and B).

### 3.2. Expression of subsets of BART miRNAs in tumor xenografts and in cultured cells derived from AGS cells carrying derivatives of EBV

EBV-negative AGS gastric carcinoma cells were engineered to maintain EBV-derived plasmids that express different subsets of the BART miRNAs. The first pair of plasmids expressed either all of the BART miRNAs (3829) except for the distal BART2 or none of them (3828). AGS cells (5×10^6^ cells) carrying either plasmid were each injected subcutaneously into each of four sites into two nude mice. Palpable tumors arose at five tested sites in one month for cells with 3828 and in seven sites for cells with 3829 (Table 1) and ranged from 0.5 to almost 1 cm in diameter. These experiments were repeated with smaller inocula of 1×10^5^ cells with 16 injections for AGS cells carrying 3828 and 20 injections of AGS cells carrying 3829. Palpable tumors arose at 3.5 months in 25% of the sites injected with AGS cells carrying 3828 which lacks the BART miRNAs while 100% of the sites injected with AGS cells expressing the BART miRNAs yielded tumors (Table 1). These frequencies are statistically different and indicate that the BART miRNAs provide these xenografts a selective advantage to grow as tumors.

**Fig. 3:**
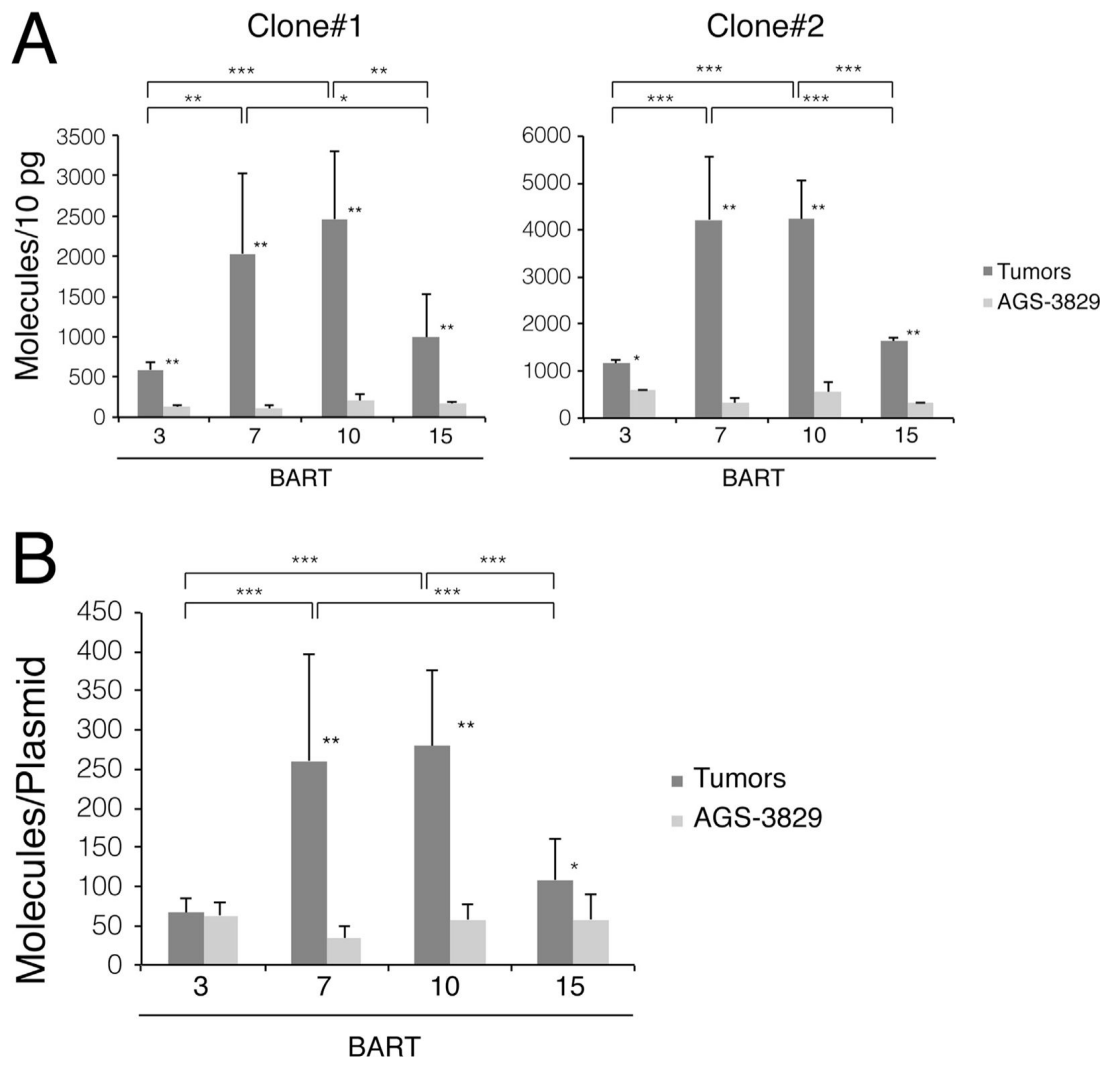
BART7 and BART10 accumulate to higher levels in AGS-3289-induced tumor xenografts than in their parental cells grown in culture. (A) 1×10^5^ cells of two clones of AGS-3829 cells were injected in parallel into mice to induce tumors as and analyzed as in Fig. 1. (B) Each BART miRNA expression level was normalized to the number of EBV genomes measured by real-time PCR with the results of 2 clones pooled. All miRNAs were measured in more than three independent experiments. *, p < 0.05; **, p < 0.005; ***, p < 0.0005.

The levels of BART miRNAs were measured in two clones of AGS cells carrying 3829 propagated in culture and as tumors in nude mice. Total RNAs were isolated and the BART miRNAs assayed by stem-loop real-time PCR. These cells in general expressed lower levels of the BART miRNAs under all conditions than SNU-719 cells but mirrored the differences found with the SNU-719 cells. Both AGS clones when grown as tumors expressed higher levels of the four BART miRNAs assayed than when grown in culture (Fig. 3A). BART miRNAs 7 and 10 accumulated to higher levels in all cells in all conditions than did BART miRNAs 3 and 15. When the levels of these miRNAs were normalized to the number of plasmid templates per AGS cells, three of the four miRNAs were expressed at higher levels in the tumors than in the cells grown in culture (Fig. 3B) (Table 3).

**Fig. 4:**
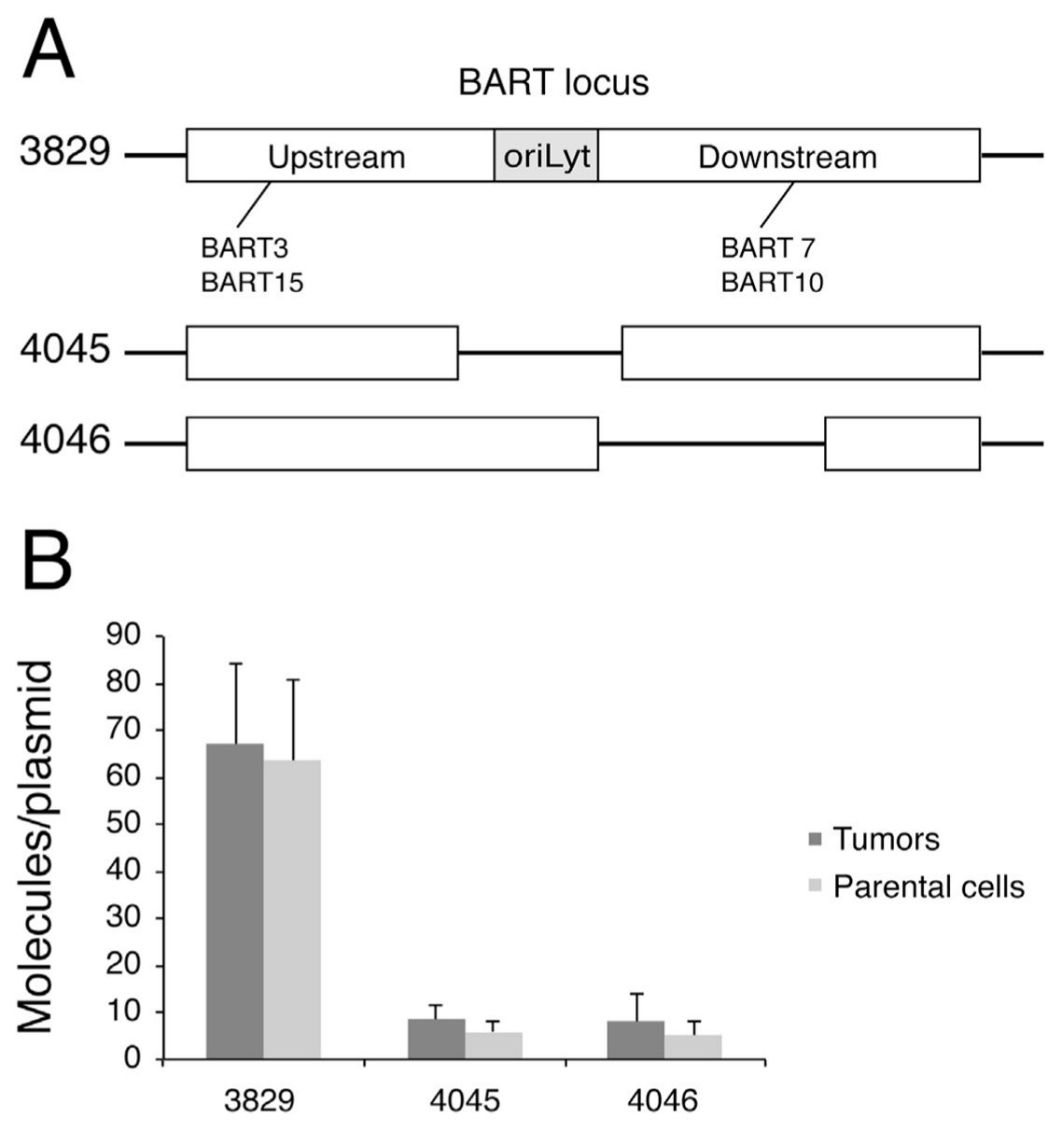
Deletions within the BART locus decrease the accumulation of BART miRNAs. (A) The plasmid 3829 and its deleted derivatives, 4045 and 4046, are depicted. Plasmid 4045 lacks *OriLyt* and plasmid 4046 lacks one-half of the downstream portion. (B) 1×10^5^ AGS-3829, AGS-4045, AGS-4046 cells were injected in parallel into mice to induce tumors. The tumors were collected and total RNA and DNA were isolated and analyzed as in Figs. 1 and 3. The level of BART3 which is expressed by all of these plasmids was measured by stem loop real-time PCR, and then normalized to the number of EBV genome. The miRNA expression per genome in at least two different clones was measured in more than three independent experiments.

**Fig. 5:**
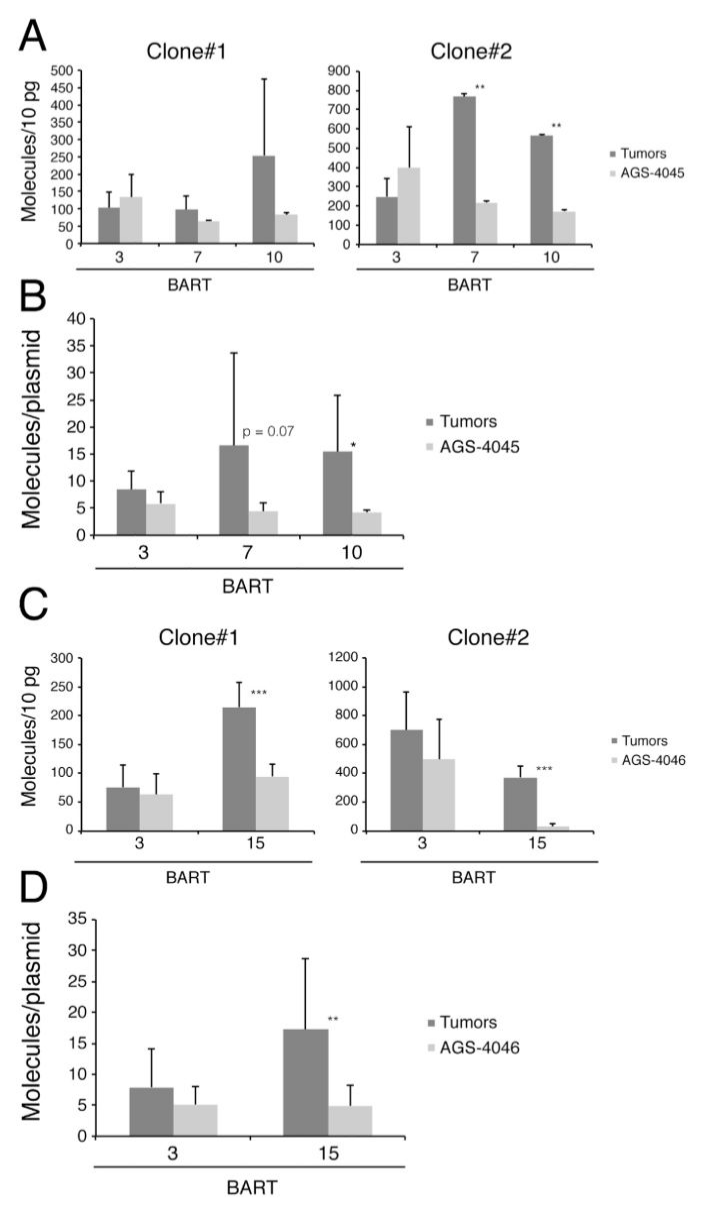
Some BART miRNAs tend to be expressed at higher levels in tumors formed by AGS-4045 and AGS-4046 than in their parental cells grown in culture. 1×10^5^ cells of two clones of AGS-4045 and AGS-4046 cells were injected in parallel into mice to induce tumors. The tumors were collected and total RNA (A and C) and DNA (B and D) were isolated and analyzed as in Figs. 1 and 3. All miRNAs were measured in more than three independent experiments. *, p < 0.05; **, p < 0.005; ***, p < 0.0005.

All of these experiments showed that the BART miRNAs in the portion upstream of oriLyt, 3 and 15, accumulated to lower levels than did 7 and 10 encoded downstream of oriLyt. We therefore asked if *OriLyt* which divides these two portions (Fig. 4A) differentially regulated the expression of these sets of miRNAs. OriLyt cannot function to support DNA synthesis on these plasmids because the required viral genes are not present. It is a genetically complex element, though, and could recruit transcription factors that regulate the expression of the viral miRNAs. We also asked if deleting part of the BART would allow a more efficient accumulation of the mRNAs encoded upstream oriLyt. To address these questions, two deleted variants of the plasmid 3829 were made which lacked *OriLyt* (4045) or lacked one-half of the downstream portion (4046) and introduced into AGS cells. Two clones of these cells carrying each plasmid were selected and both grown in culture or injected into nude mice with inocula of 1×10^5^ cells in 8 or 24 sites. These inocula yielded palpable tumors for 75% of the injections in 3.5 months (Table 1). The relevant miRNAs were assayed in these cells grown in culture or in the tumors that formed as xenografts. Plasmid 4045 encodes BART 3, 7, and 10 while plasmid 4046 encodes BART 3 and 15. All of these clones in all conditions expressed lower levels of all of these miRNAs (Fig. 4B) than did the SNU-719 or the AGS cells carrying the intact BART locus (3829). Again the levels of expression of several of the miRNAs were higher in the tumors than in the cells grown in culture (Fig. 4A and C). The varied levels of expression persisted with these derivatives with BART 7 and 10 trending to higher levels than did BART 3 in the tumors but not in the cells grown in culture (Fig. 5A). Neither the deletion of OriLyt nor of one-half of the DNA encoding the downstream portion of the transcript led to an increased accumulation of the remaining encoded miRNAs (Fig. 5A and C). When these levels were normalized to the numbers of plasmids templates per cell (Fig. 5B and D) ( Tables 4 and 5), the values were significantly lower than those found in SNU-719 cells and in AGS cells carrying the intact BART locus. These analyses demonstrated that the integrity of the BART locus is needed for the efficient expression of BART miRNAs and that while *OriLyt* may act in *cis* to regulate that expression other regions within the locus are needed for optimal expression, too.

## 4. Discussion

EBV’s BART miRNAs have been shown to provide the virus selective advantages on infecting primary B-cells, to normal and tumor cells grown in culture, and to tumors grown as xenografts in immuno-compromised mice. For example, the BART miRNAs have been found to inhibit both CD4- and CD8-mediated immune responses to infected cells (Albanese et al., 2016; Feederle et al., 2011; Lin et al., 2015; Tagawa et al., 2016), to foster tumor cell survival in culture (Vereide et al., 2014), and enhance tumor growth in vivo (Qiu et al., 2015). Biopsies of carcinomas have been found to express unexpectedly high levels of these miRNAs as have carcinomas grown as xenografts (Chen et al., 2010; Qiu et al., 2015). In the latter studies the same cells grown in culture expressed lower levels of the miRNAs than did the tumors. We have analyzed multiple cell types grown both in culture and as tumor xenografts in nude mice to elucidate the mechanism underlying the higher level of expression of BART miRNAs found in tumors.

These analyses show that the different BART miRNAs accumulate in cells to different levels. Those in the portion of the transcript downstream of oriLyt, in particular BART 7 and 10, accumulate to higher levels than do miRNAs 3 and 15 encoded in upstream of oriLyt. This disparity is particularly apparent in tumors (Figs. 1–3 and 5) but recapitulates similar measurements made in multiple studies in various cells in culture (Pratt et al., 2009; Qiu et al., 2015). Normalizing the levels of the BART miRNAs to the number of plasmid templates per cell reduces the magnitude of the levels of expression so that the BART miRNAs encoded in the upstream portion (Fig. 4) were expressed similarly in both tumors and in cells grown in culture. The BART miRNAs encoded in the portion downstream of oriLyt were expressed at 10–20-fold higher levels in tumors than in cells in culture prior to normalization while these differences dropped to 3–8-fold after normalization. These normalized measurements reveal two facets of the regulation of EBV’s miRNAs. First the high levels of the BART miRNAs detected in SNU-719 tumors reflect in part high numbers of the viral templates encoding them. Second because the BART miRNAs provide tumor cells selective advantages when grown as xenografts (Qiu et al., 2015) (Table 1, this study), those that accumulate to high levels are candidates for better mediating those advantages.

The higher levels of some of the BART miRNAs measured per DNA template in tumor cells could arise from alterations in their rate of transcription, in miRNA processing, and/or in their stability. Comparisons of the expression of the highly expressed BART miRNAs 7 and 10 in tumors of SNU-719, AGS carrying 3829, and AGS carrying 4045 cells show that the number of these miRNAs per DNA template were about 1000, 250, and 15 respectively (Figs. 2B, 3B, and 5B). This 60-fold range in specific miRNAs per template is consistent both with their rate of transcription differing in these cells and the deletion of the *OriLyt* locus in the plasmid 4045 decreasing this rate. It is also likely that the BART miRNAs 7 and 10 are intrinsically stable because Kang et al. (2015) who expressed them individually from lentiviral vectors in AGS cells found them to accumulate efficiently too. Our analyses show that the EBV’s BART miRNAs are expressed at relatively high levels in tumors both because viral genomes and their miRNA transcripts accumulate to higher levels than in the corresponding cells grown in culture.

## Figures and Tables

**Table 1:** Cell type used for mouse injection, tumor growth number and frequency are shown.

Cell Type	Tumor Growth	Total Injections	Tumor Growth Frequency	
5*10^6 cells/per injection				
SNU-719	8	8	100 %	
AGS-NC	5	8	62.5 %	
AGS-3829	7	8	87.5 %	
1*10^5 cells/per injection				
SNU-719	8	8	100 %	
AGS-NC	4	16	25 %	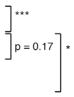
AGS-3829	20	20	100 %	
AGS-4045	6	8	75 %	
AGS-4046	18	24	75 %	

* , p < 0.05;

*** , p < 0.0005.

**Table 2:** EBV genome copy number in SNU-719-induced tumors and in parental cells.

	SNU-719/Tumors	SNU-719
Genome copy number/cell (5*10^6 per injection)	112.2 ± 9.4	39.7 ± 5.4
Genome copy number/cell (1*10^5 per injection)	206.8 ± 120.4	94.2 ± 65.5
Average	172.8 ± 103.8	69.4 ± 54.5

**Table 3:** Plasmid copy number in AGS-3829-induced tumors and in parental cells.

	AGS-3829/Tumors	AGS-3829
Plasmid copy number/cell (Clone#1)	11.2 ± 2.0	7.4 ± 4.9
Plasmid copy number/cell (Clone#2)	12.0 ± 2.0	15.1 ± 6.0

**Table 4:** Plasmid copy number in AGS-4045-induced tumors and in parental cells.

	AGS-4045/Tumors	AGS-4045
Plasmid copy number/cell (Clone#1)	18.9 ± 16.9	19.8 ± 1.2
Plasmid copy number/cell (Clone#2)	16.4 ± 6.7	32.8 ± 6.8

**Table 5:** Plasmid copy number in AGS-4046-induced tumors and in parental cells.

	AGS-4046/Tumors	AGS-4046
Plasmid copy number/cell (Clone#1)	19.5 ± 2.5	20.7 ± 13.4
Plasmid copy number/Cell (Clone#2)	19.9 ± 8.5	18.6 ± 10.4
